# Impact of IGFBP-3 A-202C genetic variant on breast cancer susceptibility and serum biomarkers (IGFBP-3 and IGF-1) in Palestinian women

**DOI:** 10.1371/journal.pone.0325289

**Published:** 2025-06-10

**Authors:** Heba Mohammed Arafat, Tengku Ahmad Damitri Al-Astani, Noorazliyana Shafii, Rosediani Muhamad, Ohood Mohammed Shamallakh, Ihab Naser, Nahed Al Laham, Alaa Siddig

**Affiliations:** 1 Department of Chemical Pathology, School of Medical Sciences, Health Campus, Universiti Sains Malaysia, Kubang Kerian, Kelantan, Malaysia; 2 Department of Family Medicine, School of Medical Sciences, Health Campus, Universiti Sains Malaysia, Kubang Kerian, Kelantan, Malaysia; 3 Department of Medical Laboratory Sciences, Faculty of Health Sciences, Islamic University of Gaza, Gaza City, Palestine; 4 Department of Clinical Nutrition, Faculty of Applied Medical Sciences, Al-Azhar University- Gaza, Gaza City, Palestine; 5 Department of Laboratory Medicine, Al Azhar University-Gaza, Gaza City, Palestine; 6 Department of Pathology, School of Medical Sciences, Universiti Sains Malaysia, Kelantan, Malaysia; ICMR: Indian Council of Medical Research, INDIA

## Abstract

Breast cancer is the most common diagnosed cancer and the leading cause of cancer-related deaths among women globally. This study examined the impact of insulin-like growth factor binding protein-3 (IGFBP-3) A-202C polymorphism (rs2854744) on breast cancer risk and its association with insulin-like growth factor-1 (IGF-1) and IGFBP-3 serum levels among Palestinian women in the Gaza Strip. Understanding these genetic variants could guide the development of early detection strategies and personalized interventions for breast cancer in underrepresented populations. A case-control study involved 112 women with newly diagnosed breast cancer and 222 healthy controls. Genotyping of the IGFBP-3 A-202C polymorphism was performed using polymerase chain reaction and restriction fragment length polymorphism (PCR-RFLP), and serum IGFBP-3 and IGF-1 levels were measured. The CC genotype of IGFBP-3 A-202C was observed in 70.5% of cases and 20.7% of controls, conferring a 16-fold increased breast cancer risk (OR=16.237; 95%CI 7.904, 33.356, *p* ≤ 0.001). The AC genotype was found in 19.6% of cases and 32.4% of controls, also associated with ~ 3-fold increased risk (OR=2.889; 95%CI 1.319, 6.325, *p* = 0.008). The CC genotype conferred a 28-fold increased risk in premenopausal women (OR=28.050; 95%CI 10.281, 76.527, *p* ≤ 0.001) and 5-fold in postmenopausal women (OR=5.333; 95%CI 1.711, 16.620, *p* = 0.004). Women with the CC genotype had the highest mean serum IGF-1 levels (116.49 ng/mL, *p* ≤ 0.001) and IGFBP-3 levels (3.76 µg/mL, *p* = 0.006) compared to those with the AA genotype. Positive correlations were observed between IGFBP-3 polymorphism and serum IGF-1 (*r*_*s* _= 0.175, *p* ≤ 0.001) and IGFBP-3 levels (*r*_*s*_ = 0.164, *p* = 0.003). The IGFBP-3 A-202C polymorphism, especially the CC genotype, is strongly associated with elevated serum IGFBP-3 and IGF-1 levels and increased breast cancer risk, highlighting its potential as a biomarker for breast cancer screening and risk stratification. Further studies are needed to validate these findings in diverse populations.

## Introduction

Cancer is the second leading cause of death globally, with 20 million new cases and 9.7 million deaths in 2022 [1,2]. Breast cancer remains the most diagnosed malignancy in women, accounting for 2.3 million new cases and 685,000 deaths worldwide [3,4]. In Palestine, it is the most prevalent cancer among women, with 934 new cases reported in 2022, comprising 36.9% of female cancer diagnoses and the leading cause of cancer-related mortality [5]. In the Gaza Strip, 394 newly registered cases of breast cancer represent 19.2% of new cancer cases. Among females, it comprises 36.9% of all cancer diagnoses and is the first leading cause of death [5,6].

Insulin-like growth factor-1 (IGF-1) is a biomarker linked to cancer risk and mortality [7]. IGF-1 promotes cell growth and inhibits apoptosis, processes that contribute to tumor formation [7]. IGFBP-3, its primary binding protein, regulates these effects and plays a dual role in either promoting or inhibiting cancer progression [8,9]. IGF-1 and its binding proteins and receptors play critical roles in human tissue development, particularly in the mammary gland, where dysregulation can increase breast cancer risk and accelerate its progression [10]. IGFBP-3 also exhibits IGF-independent effects, such as inhibiting cell growth and inducing apoptosis, and can interact with breast cancer in both stimulatory and inhibitory manners [11,12]. Studies have shown that IGFBP-3 and IGF-1 levels are related to the development and recurrence of breast cancer [13].

Single nucleotide polymorphisms (SNPs) are the most prevalent genetic variations among individuals and have shown associations with clinical traits such as cancer risk, making them potential biomarkers for assessing risk and screening for cancer [14,15]. Genetic polymorphisms within the IGFBP-3 gene can modulate circulating IGFBP-3 levels and impact a person’s vulnerability to different carcinomas, including breast cancer. Among the IGFBP-3 functional polymorphisms found in IGFBP-3, the promoter rs2854744 polymorphism (A-202C) is the most significant and extensively researched [16]. It involves a single nucleotide change from adenine to cytosine (A > C) at the −202 locus, resulting in AA, AC, and CC genotypes. The IGFBP-3 polymorphism can influence IGFBP-3 transcriptional levels, regulating the availability and function of IGF-1 [17].

Despite numerous investigations collectively indicating a link between the IGFBP-3 A-202C polymorphism and the risk of developing breast cancer, the results are still not conclusive. Some studies have suggested a protective role for the A allele, while others have shown an increased risk associated with the C allele. For instance, a large case-control study in the UK, including 4,647 breast cancer cases and 4,564 controls, found that the AA genotype of IGFBP-3 rs2854744 was associated with increased circulating IGFBP-3 levels and a modestly reduced risk of breast cancer (OR = 0.87; 95% CI 0.77, 0.99, p = 0.03) [18]. In contrast, a case-control study of 2,503 Chinese women in Shanghai found that individuals carrying the C allele of the IGFBP-3 A-202C polymorphism had a 1.6-fold increased risk of developing breast cancer [19]. These differences in findings may be due to variations in the study populations, genetic backgrounds, environmental factors, and methodologies, highlighting the need for further research to clarify the role of this SNP in breast cancer risk. In addition, multiple studies have failed to establish a clear relationship between the A-202C polymorphism and breast cancer incidence, further complicating the interpretation of this genetic variant’s role [20–22]. These conflicting findings highlight the need for population-specific studies to better understand the relationship between IGFBP-3 polymorphisms and breast cancer risk.

This study aims to address these contradictions by focusing on Palestinian women, a population underrepresented in global research. By examining the IGFBP-3 A-202C polymorphism in the context of both IGFBP-3 and IGF-1 serum levels, we clarify the role of this polymorphism as a biomarker for early breast cancer detection, particularly in Palestinian women. Understanding these relationships may help identify women who are more susceptible to develop breast cancer, which is crucial for developing strategies for early diagnosis and effective therapies to mitigate breast cancer risk. The study’s focus on Palestinian women addresses a critical gap in breast cancer research, as data on the IGFBP-3 A-202C polymorphism in Middle Eastern populations are scarce. By exploring this genetic variant and integrating serum biomarkers, the research provides unique insights into genetic and serum biomarkers as risk factors in an understudied group. This contributes to understanding global breast cancer heterogeneity and highlights the importance of population-specific studies for tailored prevention and treatment strategies. The study’s findings on the IGFBP-3 A-202C polymorphism in Palestinian women in the Gaza Strip highlight potential population-specific genetic, and biochemical influences on breast cancer risk. Comparisons with other populations, such as Caucasian or Asian groups, could reveal unique patterns, emphasizing the need for population-specific research. This polymorphism could be integrated into targeted genetic screening programs for Palestinian women, advancing personalized breast cancer prevention. The inclusion of serum biomarkers further enhances the clinical relevance of these findings by providing a more comprehensive risk assessment. Evidence regarding IGFBP-3 gene polymorphisms’ implications in serum levels of IGFBP-3 and IGF-1 in the Gaza Strip and their association with breast cancer risk remains unclear. To the best of our knowledge, this is the first study to elucidate the role of IGFBP-3 A-202C (rs2854744) gene polymorphism and IGF-1 and IGFBP-3 serum levels as biomarkers for early detection of breast cancer among Palestinian women in the Gaza Strip offering insights into its potential as a biomarker for regional breast cancer risk.

## Materials and methods

### Study design and settings

This case-control study was carried out between January 2021 and April 2023 in governmental hospitals with oncology departments in the Gaza Strip, specifically Al-Shifa Hospital and Turkish Palestinian Friendship Hospital for cases, and in Al-Remal Clinic for controls. Palestinian women were chosen as the study population to address the gap in genetic research in underrepresented regions, particularly in the context of breast cancer susceptibility. Cases and controls were matched by age to minimize confounding effects related to age-associated breast cancer risk. The sample size was calculated using G*Power, and after accounting for a 10% non-response rate and a 1:2 ratio of case to control was used to enhance statistical power and detect genotype effects in a limited sample size, the final sample size was 336, comprising of 112 newly diagnosed breast cancer women, and 224 healthy controls.

### Study participants

Newly diagnosed women with breast cancer, confirmed with histopathological examination, were selected from the computerized medical records at the aforementioned governmental hospitals as cases. Healthy volunteers without breast cancer, whose screening mammography or breast ultrasound imaging results were negative, were selected as controls from the electronic records at Al-Remal Clinic.

### Eligibility criteria

The cases were women aged 25–65 years with newly diagnosed breast cancer, confirmed by histopathology, who were not undergoing any medical treatment (such as radiation, targeted therapy, chemotherapy, hormone therapy, or alternative therapy). Controls were healthy, age-matched women, with negative mammogram screenings (aged 40–65 years) or negative breast ultrasound imaging (aged 25–39 years). Cases were excluded if they were immunocompromised or immunosuppressed, had diabetes mellitus, thyroid disorders, organ failure, or acute infections within the past month. Conversely, controls were excluded if they were breastfeeding, had a history of malignancy, known medical conditions, acute infections within the past month, or current breast pathology or lesions.

### Study tool and data collection

Participants were recruited using convenience sampling. Breast cancer cases and healthy controls were interviewed after obtaining informed consent, using a structured and validated face-to-face questionnaire. Biochemical analyses of serum IGF-1 and IGFBP-3, as well as genetic analysis of the IGFBP-3 A-202C, were conducted for both cases and controls.

The Helsinki Committee provided its ethical permission (approved number PHRC/HC/699/20) for the study project in the Gaza Strip during its meeting on February 3, 2020. Further ethical approval was received on January 13, 2022, from the USM Human Research Ethics Committee with the JEPeM USM Code: USM/JEPeM/20020122. Informed consent was obtained from all participants before their recruitment into the study. To ensure participant privacy and confidentiality, interviews were conducted in a private room, separate from clinical areas, by trained research staff. Participants were informed that their participation was voluntary and their responses would be kept strictly confidential. All data collected were anonymized by assigning a unique study ID to each participant, and all identifying information was removed before data analysis. Data were stored securely on password-protected computers and accessed only by authorized research personnel.

Detailed procedures for blood sample collection and biochemical analysis have been documented previously [23]. Briefly, fasting blood samples were obtained from participants at Al-Shifa Hospital, Turkish Palestinian Friendship Hospital, and Al-Remal Clinic. Samples were delivered to the Palestinian Medical Relief Society under cold-chain conditions (4°C) within 24 hours to ensured quality-controlled conditions. Upon collection, three ml were placed in plain red-top tubes and centrifuged for 10 minutes at 3000 revolutions per minute (rpm). The serum was promptly separated and analysed for biochemical analysis of IGFBP-3 and IGF-1, using an auto-chemiluminescence immunoassay analyzer from the MAGLUMI 800 series (Snibe, China) following the manufacturer’s protocols. The blood was only drawn once, and the remaining serum samples were securely stored at −20°C until the completion of the study. At that point, they were destroyed according to institutional biohazard waste disposal protocols. Participation in the study lasted from the time participants were enrolled until they received the study’s findings.

### Genomic DNA analysis

For genomic DNA analysis, three ml of the obtained whole blood were placed in sterile ethylene diamine tetraacetic acid (EDTA) tubes and were transferred to the Genetics Laboratory at Al-Aqsa University. DNA extraction was performed with strict adherence to manufacturer protocols of the blood using the Wizard Genomic DNA Purification Kit (Promega, USA). All reagents were prepared fresh, and a blank control was included in every PCR run to ensure the absence of contamination. Concentration and purity were assessed via NanoDrop spectrophotometry. To ensure accuracy and reproducibility in genotyping, strict quality control measures were implemented. These included duplicate testing for ambiguous results and the inclusion of positive and negative controls during PCR amplification and restriction digestion. PCR components used in this study are presented in (Table 1 and 2).

**Table 1:** Conventional PCR mixture used in this study.

Components	Initial concentration	Volume for 1 reaction (µl)	Final concentration
**Forward primer**	10 µM	1	1 µM
**Reverse primer**	10 µM	1	1 µM
**GoTaq® Green Master Mix, 2X**	–	12.5	–
**PCR grade water**	–	8.5	–
**Template DNA**	50 ng	2	100 ng/µl
**Total**	–	25	–

**Table 2:** PCR-RFLP mixture used in this study.

Components	Volume for 1 reaction (µl)
**Fast digest enzyme (*Alw21I*)**	1 µl
**10X Fast digest buffer**	2 µl
**H**_**2**_ **O**	7 µl
**PCR product**	10 µl
**Total**	20 µl

**Fig 1:**
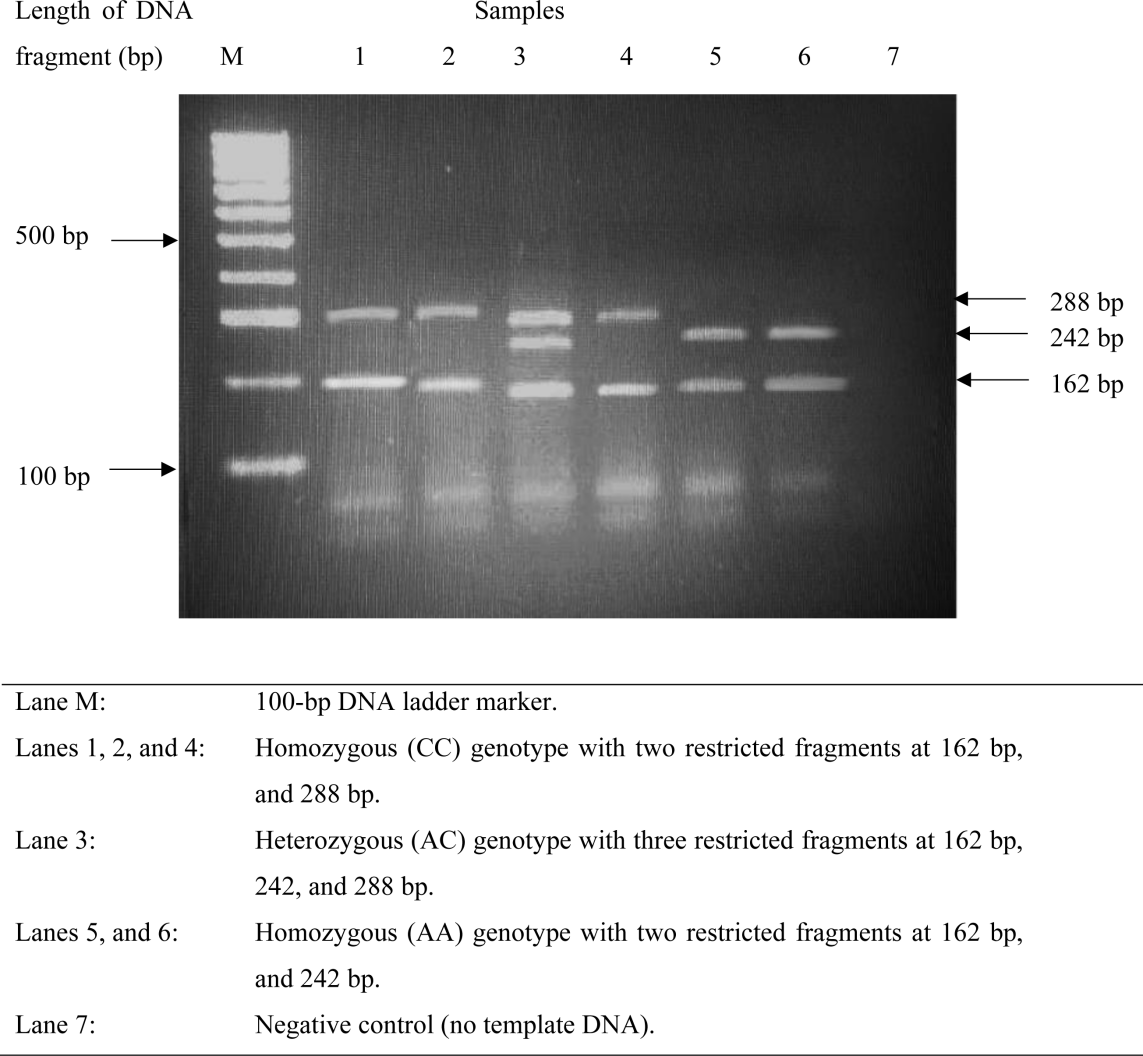
Representative gel electrophoresis results for IGFBP-3 A-202C genotyping after digestion with the *Alw21I* restriction enzyme.

Insulin-like growth factor binding protein-3 A-202C (rs2854744) SNP genotyping was carried out using the PCR-RFLP method. The IGFBP-3 promoter region’s 244-bp fragment containing the A to C polymorphic site was amplified utilizing the subsequent particular forward (5’-CCACGAGGTACACACGAATG-3’) and reverse (5’-AGCCGCAGTGCTCGCATCTGG-3’) primers, designed based on a published study [24]. A 25 μl reaction mixture was used for the PCR amplification process. The following were the PCR conditions: initial denaturation at 95°C for 5 minutes, followed by 37 cycles of 95°C for 30 seconds (denaturation), 60°C for 30 seconds (annealing), and 72°C for 30 seconds (extension), with a final extension step at 72°C for 5 minutes. The PCR products were digested with the restriction enzyme *Alw21I* (Thermo Fisher Scientific, USA) and analyzed using 2% agarose gel electrophoresis stained with ethidium bromide. Electrophoresis was conducted at 80V for 60 minutes, and DNA bands were visualized under UV light using a gel documentation system. A 100-bp molecular ladder was included in each gel to determine fragment sizes. Allele identification was performed based on the presence of specific fragments: homozygous AA (242 bp and 162 bp), homozygous CC (288 and 162 bp), and the heterozygous AC (288, 242, and 162 bp) (Fig. 1).

### Statistical analysis

Data analysis was performed using IBM SPSS Statistics version 28 (IBM Corp., Armonk, NY, USA). After entering and cleaning the data, statistical significance was set at *p*-values ≤ 0.05. With a sample size of 334, the data were considered normally distributed. For SNP analyses, genotype frequencies and associations with circulating levels were calculated for all participants and stratified by menopausal status. The genotype distributions between healthy controls and breast cancer cases, as well as by menopausal status were compared using the Chi-squared test. Logistic regression was applied to estimate the breast cancer risk (Odds Ratios (OR) and 95% Confidence Intervals (CIs)) associated with different genotypes (homozygous AA, homozygous CC, and heterozygous AC) in matched case-control sets and stratified by menopausal status. Spearman’s rank correlation test was used to explore relationships between the IGFBP-3 A-202C genotype and serum levels of IGFBP-3 and IGF-1 among cases and controls. Additionally, independent t-tests were used to compare mean serum levels of IGFBP-3 and IGF-1 stratified by IGFBP-3 genotype.

### Study flowchart

**Figure:**
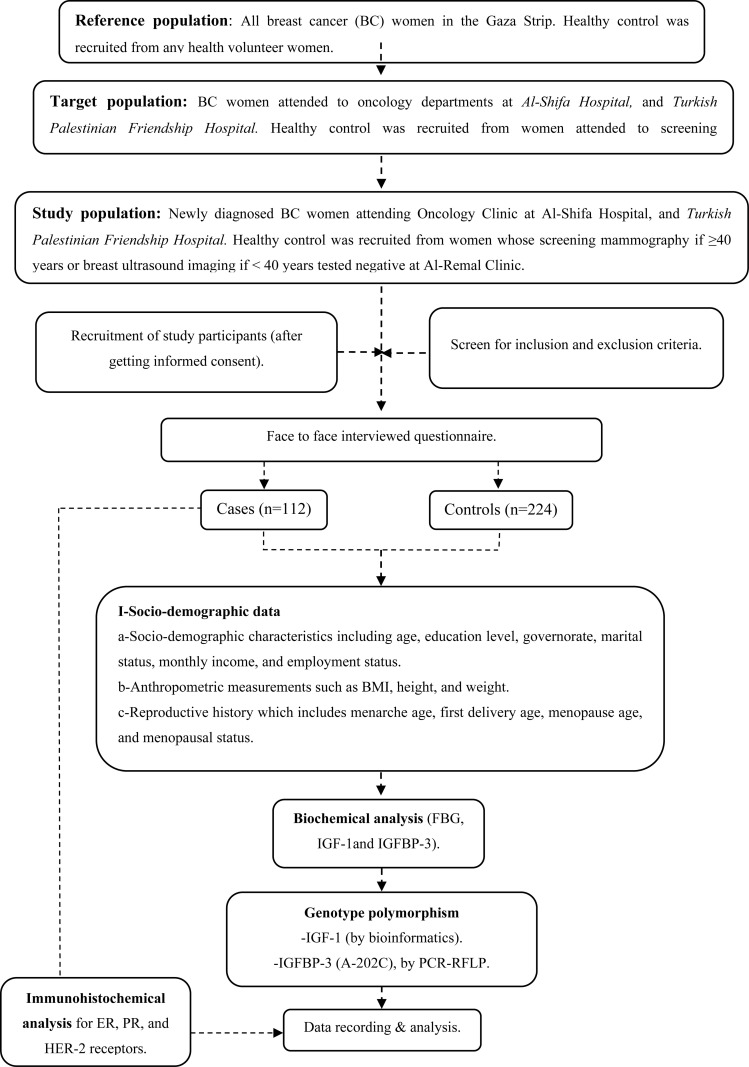


## Results

### Genotype distribution among study participants and the relationship between the IGFBP-3 A-202C polymorphism and the risk of breast cancer

The analysis of the IGFBP-3 A-202C polymorphism’s gene distribution in 112 newly diagnosed women with breast cancer and 222 healthy controls was conducted. Only 9.8% of breast cancer cases were homozygous for the A allele, compared to 46.8% of controls. Heterozygous AC genotypes were observed in 19.6% of breast cancer cases and 32.4% of controls. The C allele was notably prevalent in breast cancer cases, with 70.5% being homozygous for the C allele versus 20.7% of controls, indicating a significant association between the C allele and increased breast cancer risk (*p* ≤ 0.001). Further analysis showed that women with the CC genotype of IGFBP-3 A-202C had a 16-fold higher risk of breast cancer compared to those with other genotypes (OR=16.237; 95%CI 7.904, 33.356, *p* ≤ 0.001). Those with the heterozygous AC genotype were approximately 3 times more likely to develop breast cancer compared to those with AA genotypes (OR=2.889; 95%CI 1.319, 6.325, *p *= 0.008). These results underscore the strong relationship between the C allele of the IGFBP-3 A-202C polymorphism and breast cancer susceptibility (Table 3).

**Table 3:** Genotype distribution and breast cancer risk for IGFBP-3 A-202C polymorphism.

Polymorphism	Genotypes	Cases N = 112	Controls N = 222	χ 2 (df), *p*-value	Crude OR (95%CI)	*p*-value
		*n* (%)	*n* (%)			
**IGFBP-3 A - 202C**	**AA**	11 (9.8)	104 (46.8)	83.327 (2), ≤ 0.001	Ref.	
	**AC**	22 (19.6)	72 (32.4)		2.889 (1.319, 6.325)	0.008
	**CC**	79 (70.5)	46 (20.7)		16.237 (7.904, 33.356)	≤ 0.001

*Significant at the level of 0.05.

Note. IGFBP-3 = Insulin-like growth factor binding protein-3; *n* = Frequency; χ² (df) = Chi-square test (degrees of freedom); OR= Odds ratio; CI = Confidence interval.

### Distribution of genotypes and relationship between IGFBP-3 A-202C polymorphism and breast cancer risk stratified by menopausal status

The study examined the distribution of IGFBP-3 A-202C genotypes among 112 breast cancer cases and 222 healthy controls, stratified by menopausal status. Significant differences were found in both premenopausal (*p* ≤ 0.001) and postmenopausal groups (*p* = 0.003) indicating a strong association between the C allele and increased breast cancer risk. In premenopausal women, homozygous CC carriers were 28 times more likely to develop breast cancer compared to AA or AC carriers (OR=28.050; 95%CI 10.281, 76.527, *p* ≤ 0.001). Heterozygous AC carriers were nearly 5-fold higher risk (OR=4.552; 95%CI 1.569, 13.204, *p* = 0.005). Among postmenopausal women, the risk of breast cancer was also elevated for homozygous C carriers (OR=5.333; 95%CI 1.711, 16.620, *p* = 0.004), although the association was weaker than in premenopausal women. These findings suggest a strong correlation between the IGFBP-3 A-202C polymorphism and breast cancer susceptibility, particularly in premenopausal women (Table 4).

**Table 4:** The association between IGFBP-3 A-202C genotype among postmenopausal and premenopausal.

Genotype	Premenopausal					Postmenopausal				
	Case *N *= 112	Control *N *= 222	χ² (df), *p*-value	Crude OR (95%CI)	*p*-value	Case *N *= 112	Control *N *= 222	χ² (df), *p*-value	Crude OR (95%CI)	*p*-value
		*n* (%)	*n* (%)				*n* (%)	*n* (%)		
**IGFBP-3 A-202C**										
**AA**	5 (7.0)	88 (49.4)	70.858 (2), ≤ 0.001	Ref		6 (14.6)	16 (36.4)	11.454 (2), 0.003	Ref	
**AC**	15 (21.1)	58 (32.6)		4.552 (1.569, 13.204)	0.005	7 (17.1)	14 (31.8)		1.333 (0.361, 4.918)	0.666
**CC**	51(71.8)	32 (18.0)		28.050 (10.281, 76.527)	≤ 0.001	28 (68.3)	14 (31.8)		5.333 (1.711, 16.620)	0.004

*Significant at the level of 0.05.

Note. IGFBP-3 = Insulin-like growth factor binding protein-3; *n* = Frequency; χ² (df) = Chi-square test (degrees of freedom); OR= Odds ratio; CI = Confidence interval.

### IGFBP-3 A-202C genotype and serum levels of IGFBP-3 and IGF-1

The study investigated the relationship between IGFBP-3 A-202C genotypes and serum levels of IGF-1 and IGFBP-3. The results indicated that individuals with the homozygous CC genotype had the highest mean serum IGFBP-3 levels, with statistically significant differences (3.76 ± 1.45, *p* = 0.006) (Table 5). The mean of IGFBP-3 serum levels decreased with one or two copies of the A allele; however, the differences were not statistically significant (3.37 ± 1.39 for heterozygous AC and 3.30 ± 1.30 for homozygous AA, *p* > 0.05). For serum IGF-1, participants with the homozygous C allele had significantly higher mean levels (116.49 ± 62.06, *p* ≤ 0.001), while the lowest mean levels were observed in participants with the AA genotype (91.08 ± 42.94, *p* ≤ 0.001). These findings suggest that the C allele is associated with elevated serum levels of IGFBP-3 and IGF-1, which may have biological implications in cancer progression. Higher IGF-1 and IGFBP-3 levels in CC genotype carriers could indicate enhanced tumorigenic pathways mediated through IGF signalling, potentially influencing breast cancer susceptibility and progression. This aligns with existing evidence suggesting that increased IGF-1 bioavailability promotes mitogenic and anti-apoptotic effects, contributing to tumour growth.

**Table 5:** Mean serum IGF-1 (ng/mL) and IGFBP-3 level (mg/L) by IGFBP-3 genotype A-202C among participants.

Variables		Serum IGFBP-3				Serum IGF-1			
		Mean ± SD	Mean differences (95%CI)	t statistic (df)	*p*-value	Mean ± SD	Mean differences (95%CI)	t statistic (df)	*p*-value
**IGFBP-3 A-202C (SNP rs2854744)**	**AA**	3.30 ± 1.30	0.29 (−0.01, 0.61)	1.866 (332)	0.063	91.08 ± 42.94	16.91 (5.83, 27.99)	3.005 (297.134)	≤ 0.001
	**AC**	3.37 ± 1.39	0.16 (−0.16, 0.50)	0.992 (332)	0.322	96.70 ± 51.71	7.59 (−5.35, 20.59)	1.155 (332)	0.249
	**CC**	3.76 ± 1.45	−0.43 (−0.74, 0.12)	−2.775 (332)	0.006	116.49 ± 62.06	−22.88 (−35.57, −10.20)	−3.557 (209.184)	≤ 0.001

*Significant at the level of 0.05.

Normality assumption is fulfilled.

Note. SD = Standard deviation; IGF-1 = Insulin-like growth Factor-1; IGFBP-3 = Insulin-like growth Factor binding protein-3; df = Degree of freedom.

Data were presented as mean ± SD

### The correlation between IGFBP-3 polymorphism and IGF-1 and IGFBP-3 serum levels

Spearman correlation analysis revealed the relationships between IGFBP-3 polymorphism and IGF-1 and IGFBP-3 serum levels among participants. The results showed a weak negative correlation between the AA genotype and serum IGF-1 levels (*r*_*s*_ = −0.120, *p* = 0.029), and a positive correlation for the CC genotype (*r*_*s*_ = 0.175, *p* ≤ 0.001). Similarly, the AA genotype showed a weak negative correlation with serum IGFBP-3 levels (*r*_*s*_ = −0.114, *p* = 0.037), while the CC genotype exhibited a positive correlation with IGFBP-3 serum levels (*r*_*s*_ = 0.164, *p* = 0.003).

These results imply different patterns of relationship between serum levels of IGF-1 and IGFBP-3 and IGFBP-3 polymorphism, potentially contributing to breast cancer risk (Table 6).

**Table 6:** The correlation between IGFBP-3 polymorphism and serum level of IGF-1 and IGFBP-3.

Variables	Serum IGF-1		Serum IGFBP-3	
	Total Population *N *= 334		Total Population *N *= 334	
	Corr. Coeff.	*p*-value	Corr. Coeff.	*p*-value
**Genotype IGFBP-3 A-202C**				
**AA**	−0.120*	0.029	−0.114*	0.037
**AC**	−0.062	0.262	−0.056	0.305
**CC**	0.175**	≤ 0.001	0.164**	0.003

*Significant at the level of 0.05.

**Significant at the level of 0.01.

Note. IGF-1 = Insulin-like growth factor-1; IGFBP-3 = Insulin-like growth factor binding protein-3; Corr. Coeff. = Correlation coefficient.

*r*_*s *_=* *Spearman correlation.

## Discussion

This study found a significant association between the IGFBP-3 A-202C polymorphism and breast cancer risk among Palestinian women in the Gaza Strip. Specifically, patients with the CC or AC genotype had significantly higher breast cancer risk compared to those with the AA genotype (OR = 16.237; 95%CI 7.904, 33.356, *p* ≤ 0.001 and OR = 2.889; 95%CI 1.319, 6.325, *p* = 0.008, respectively). These findings align with several other studies, although the specific odds ratios vary. For instance, Ma et al. reported a significantly elevated risk for the CC genotype risk (OR = 2.00; 95%CI 1.25, 3.21), while Ren et al. and Wagner et al. also observed increased risks associated with the C allele in their respective study populations (OR = 1.6; 95%CI 1.1, 2.4, *p* ≤ 0.05 and OR = 1.47; 95%CI 1.01, 2.15, *p* ≤ 0.05, respectively) [16,19,25]. These collective findings suggest that the IGFBP-3 A-202C polymorphism could serve as a valuable genetic marker for breast cancer susceptibility and progression.

Our study further demonstrates a similar association between the IGFBP-3 SNP rs2854744 and breast cancer risk in both pre-and post-menopausal women. Premenopausal women with the CC genotype showed a remarkably higher risk compared to those with the AA or AC genotypes (OR = 28.050; 95%CI 10.281, 76.527, *p* ≤ 0.001). Postmenopausal women with the CC genotype also exhibited an elevated risk, though to a lesser extent (OR = 5.333; 95%CI 1.711, 16.620, *p* = 0.004). While some previous research has not found significant differences in risk based on menopausal status [18], Schernhammer et al. reported a slightly decreased, though non-significant, risk for the AA genotype in premenopausal women under 50 (RR = 0.67; 95%CI 0.20, 2.20) [26]. These discrepancies may be attributable to variations in study populations, sample sizes, genetic backgrounds, lifestyle factors, or environmental factors.

Among Palestinian women in the Gaza Strip, the IGFBP-3 A-202C polymorphism was a significant predictor of the serum levels of both IGFBP-3 and IGF-1. The CC genotype was associated with the highest mean levels of both IGFBP-3 (3.76 ± 1.45 µg/mL) and IGF-1 (116.49 ± 62.06 ng/mL), followed by the AC genotype (3.37 ± 1.39 µg/mL and 96.70 ± 51.71 ng/mL, respectively), and the lowest levels in the AA genotype (3.30 ± 1.30 µg/mL and 91.08 ± 42.94 ng/mL, respectively).

The observed association between the IGFBP-3 A-202C polymorphism and breast cancer risk may be explained through its role in regulating IGF-1 bioavailability and apoptosis. IGFBP-3 is a key modulator of the IGF axis, primarily binding to IGF-1 and controlling its interaction with the IGF-1 receptor. The C allele at position −202 has been associated with altered IGFBP-3 transcription, potentially leading to increased IGF-1 levels, which, in turn, promote cell proliferation and inhibit apoptosis—both key processes in tumorigenesis [27]. Furthermore, IGFBP-3 has been reported to exert IGF-independent tumor-suppressive effects by interacting with cell surface receptors such as transmembrane protein 219 and modulating key apoptotic pathways, including tumor protein p53 and transforming growth factor-beta signaling cascades. Variants in the IGFBP-3 gene may influence these interactions, altering the protein’s ability to induce apoptosis and suppress tumor growth [28]. Given these potential mechanisms, the observed increase in breast cancer risk associated with the C allele may be mediated through both IGF-dependent and IGF-independent pathways.

Our study found a correlation between the C allele of IGFBP-3 A-202C (SNP rs2854744) with circulating IGFBP-3 levels (*r*_*s*_ = 0.164, *p* = 0.003), in which its serum levels were associated with an increased risk of breast cancer (OR = 1.154; 95%CI 0.982, 1.357, *p* < 0.25) (data not shown). This suggests a potential association between the C allele at position −202 and the risk of breast cancer. Similarly, Ren et al. found that women who were homozygous for the C allele had the highest mean blood IGFBP-3 level, and the presence of one or two copies of the A alleles caused the level to decrease gradually (*p*- trend < 0.05). A 60% elevated risk of breast cancer was found to be associated with homozygosity for the C allele in polymorphism A-202C (OR= 1.6; 95%CI 1.1, 2.4) [19]. Contrarily, previous studies have reported different associations. Jernström et al. found a correlation between the IGFBP-3 A-202C polymorphism and serum levels of IGFBP-3, with mean levels being 4390, 4130, and 3840 ng/mL for AA, AC, and CC genotypes, respectively (*p* < 0.006) [29]. Al-Zahrani et al. also reported that the A allele was associated with increased circulating IGFBP-3 levels, with mean levels of 4.316, 3.941, and 3.614 mg/L for AA, AC, and CC genotypes, respectively, and a decreased breast cancer risk (OR (AA/CC)=0.87; 95%CI 0.77, 0.99, *p* = 0.03) [18]. In addition, in women with the AA genotype at the −202 IGFBP-3 gene, Deal et al. found that the mean levels of serum IGFBP-3 increased significantly and decreased in a stepwise manner in the presence of one or two copies of the C allele [30].

Our findings also showed that the C allele of IGFBP-3 A-202C (SNP rs2854744) correlated with higher circulating IGF-1 levels (*r*_*s *_= 0.175, *p* ≤ 0.001), which were associated with an elevated likelihood of breast cancer (OR= 1.010; 95%CI, 1.006–1.015) (data not shown) [23]. This suggests that elevated serum IGF-1 might increase the risk of breast cancer. In contrast, Schernhammer et al. did not find a relationship between the IGFBP-3 A-202C genotype and total circulating IGF-1 mean levels (AA = 183 ± 9 ng/mL vs. AC = 182 ± 6 ng/mL vs. CC = 178 ± 8 ng/mL, *p* > 0.05) [26].

Using the AA genotype as a reference, our research found increased breast cancer risk for women with the AC (OR=2.889; 95%CI 1.319, 6.325, *p* = 0.008) and CC (OR=16.237; 95%CI 7.904, 33.356, *p* ≤ 0.001) genotypes. This indicates a gradient of increased risk associated with different IGFBP-3 A-202C genotypes, with the CC genotype showing the highest risk compared to the reference genotype AA. The AC genotype also presents a significant increase in breast cancer risk, though to a lesser extent than the CC genotype. These findings underscore the potential genetic influence on breast cancer susceptibility, suggesting that certain genetic variations might predispose individuals to a higher risk of developing this type of cancer.

In summary, our findings suggest that the IGFBP-3 A-202C polymorphism, particularly the C allele, may alter IGFBP-3 transcription, increasing IGF-1 availability and promoting cell proliferation and survival, key processes in tumorigenesis. Future studies should explore the molecular mechanisms underlying this association and assess gene-environment interactions that may further modulate breast cancer risk.

## Conclusion

This study provides novel insights into IGFBP-3 A-202C (rs2854744) gene polymorphism and its association with serum levels of IGFBP-3 and IGF-1, suggesting its potential as a diagnostic marker for early detection, personalized risk assessment, and targeted interventions in high-incidence breast cancer regions such as the Gaza Strip. Advancements in molecular research have enhanced the understanding of breast cancer biomarkers and improved individualized patient therapy. These findings have potential implications for genetic counselling. Specifically, the identification of the C allele as a risk factor could be incorporated into early detection strategies for women with a family history of breast cancer, allowing for more informed risk assessment and personalized screening recommendations. Additionally, the association between the C allele with elevated IGFBP-3 and IGF-1 serum levels offers a potential target for therapeutic interventions. By focusing on these factors, targeted therapies could be developed to regulate the bioavailability of IGF-1 and modulate tumorigenic processes, ultimately improving treatment outcomes for patients at higher risk.

Our findings suggest that the IGFBP-3 A-202C polymorphism is a potential biomarker for breast cancer risk in Palestinian women; however, further research should focus on validating these findings in larger, more diverse populations and exploring the cost-effectiveness of incorporating this polymorphism into existing screening programs. Future studies should investigate the functional role of the IGFBP-3 A-202C polymorphism in breast cancer biology, assess its utility in combination with other genetic markers for risk prediction, and examine its impact on IGFBP-3 and IGF-1 expression to elucidate the underlying mechanisms contributing to breast cancer development.

## Supporting information

S1_raw_images (PDF)

## Abbreviations

IGF-1: Insulin-Like Growth Factor-1
IGFBP-3: Insulin-Like Growth Factor Binding Protein 3
PCR-RFLP: Polymerase Chain Reaction and Restriction Fragment Length Polymorphism
SNP: Single Nucleotide Polymorphism
SD: Standard Deviation
CI: Confidence Interval
OR: Odds Ratio
