# Supplementary figures and images for: Impact of IGFBP-3 A-202C genetic variant on breast cancer susceptibility and serum biomarkers (IGFBP-3 and IGF-1) in Palestinian women
Source: PLoS One. 2025 Jun 10;20(6):e0325289. doi: 10.1371/journal.pone.0325289 (PMC12151368; doi:10.1371/journal.pone.0325289)

Base Pairs

M

X

X

X

X

S1

X

X

1000

900

800

700

600

500

400

300

200

100

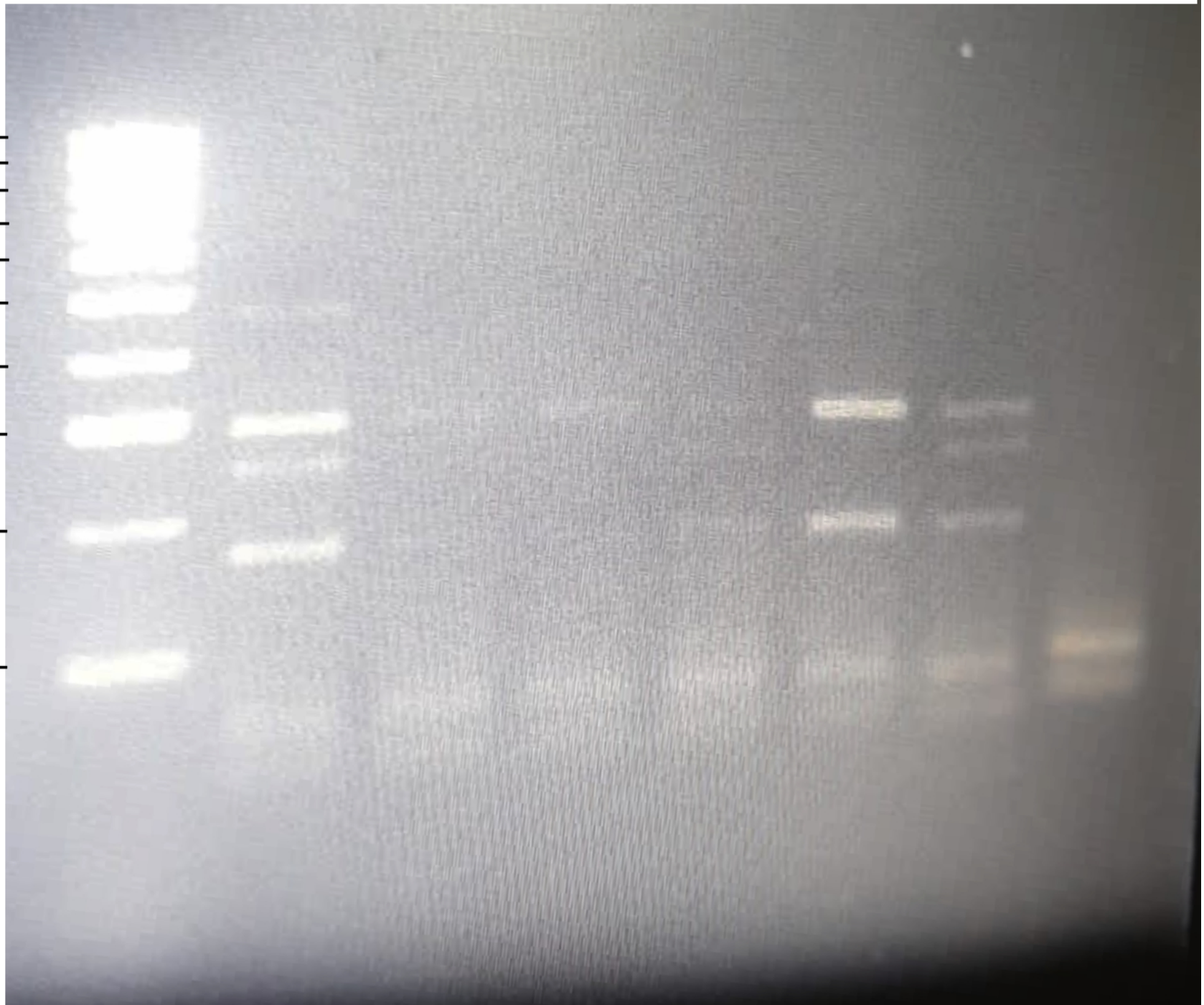

M      S6      X      X      X      X      S2      S5

Base Pairs

1000  
900  
800  
700  
600  
500  
400  
300  
200  
100

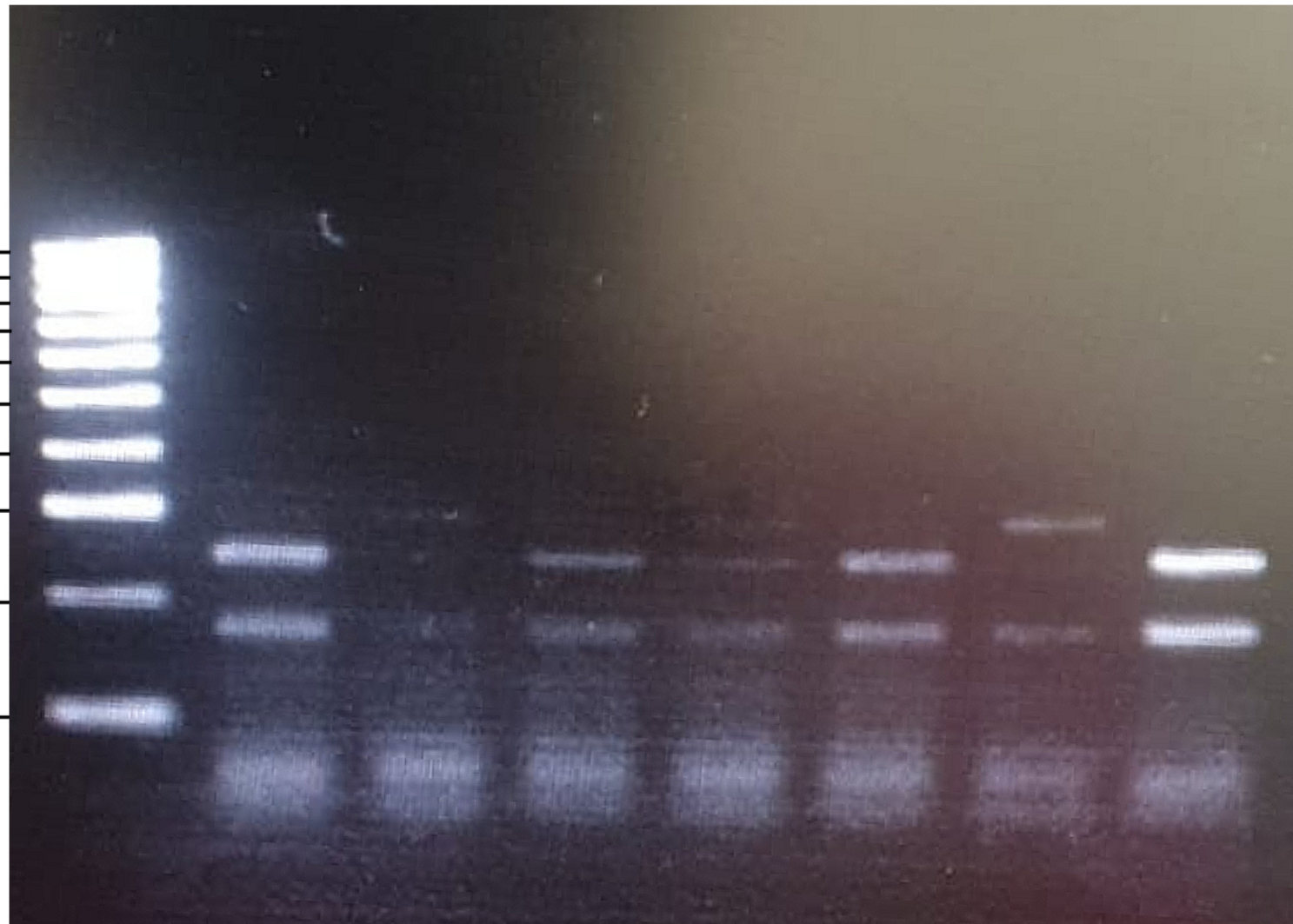

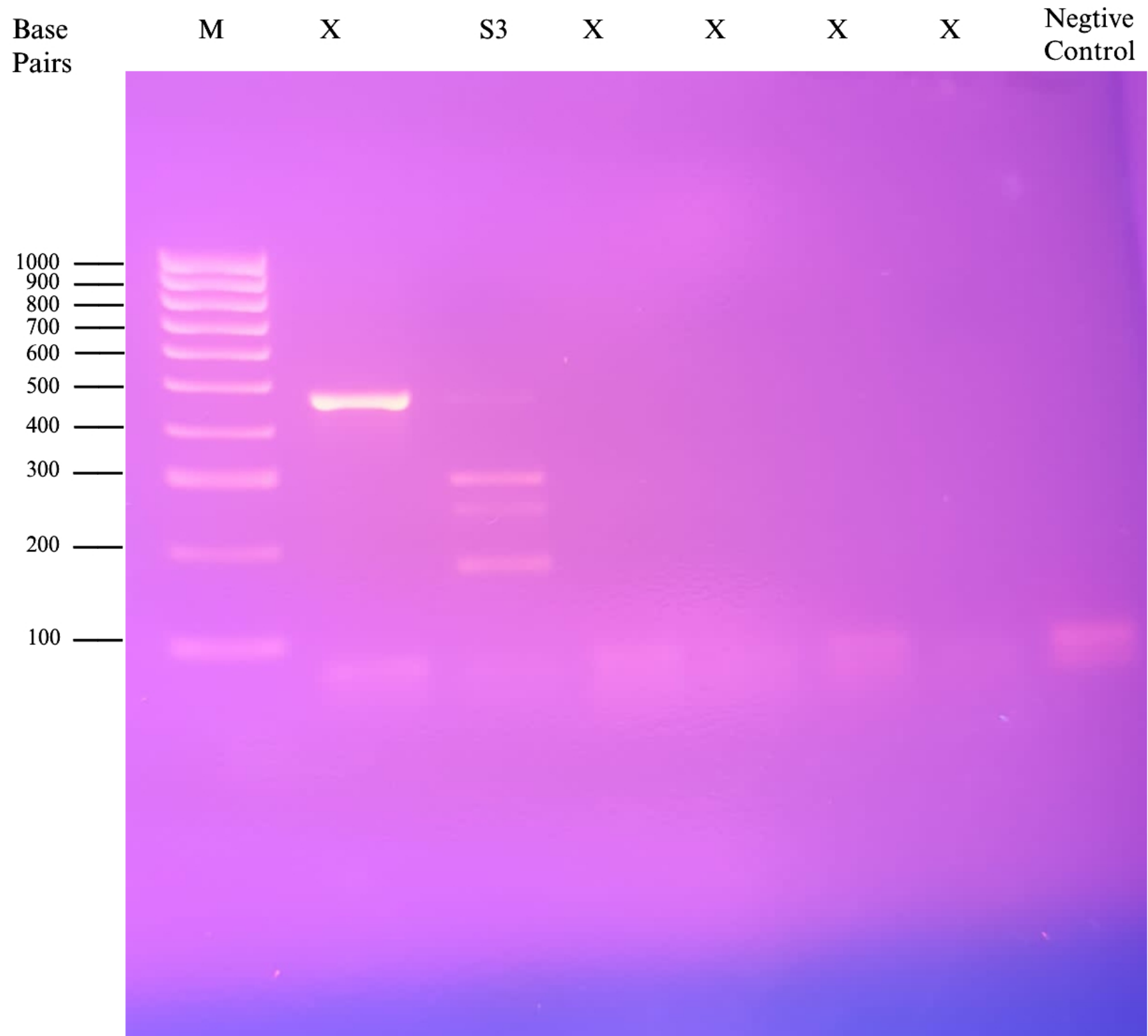

X

1000  
900  
800  
700  
600  
500  
400  
300  
200  
100

Base Pairs

M

X

X

X

1000  
900  
800  
700  
600  
500  
400  
300  
200  
100

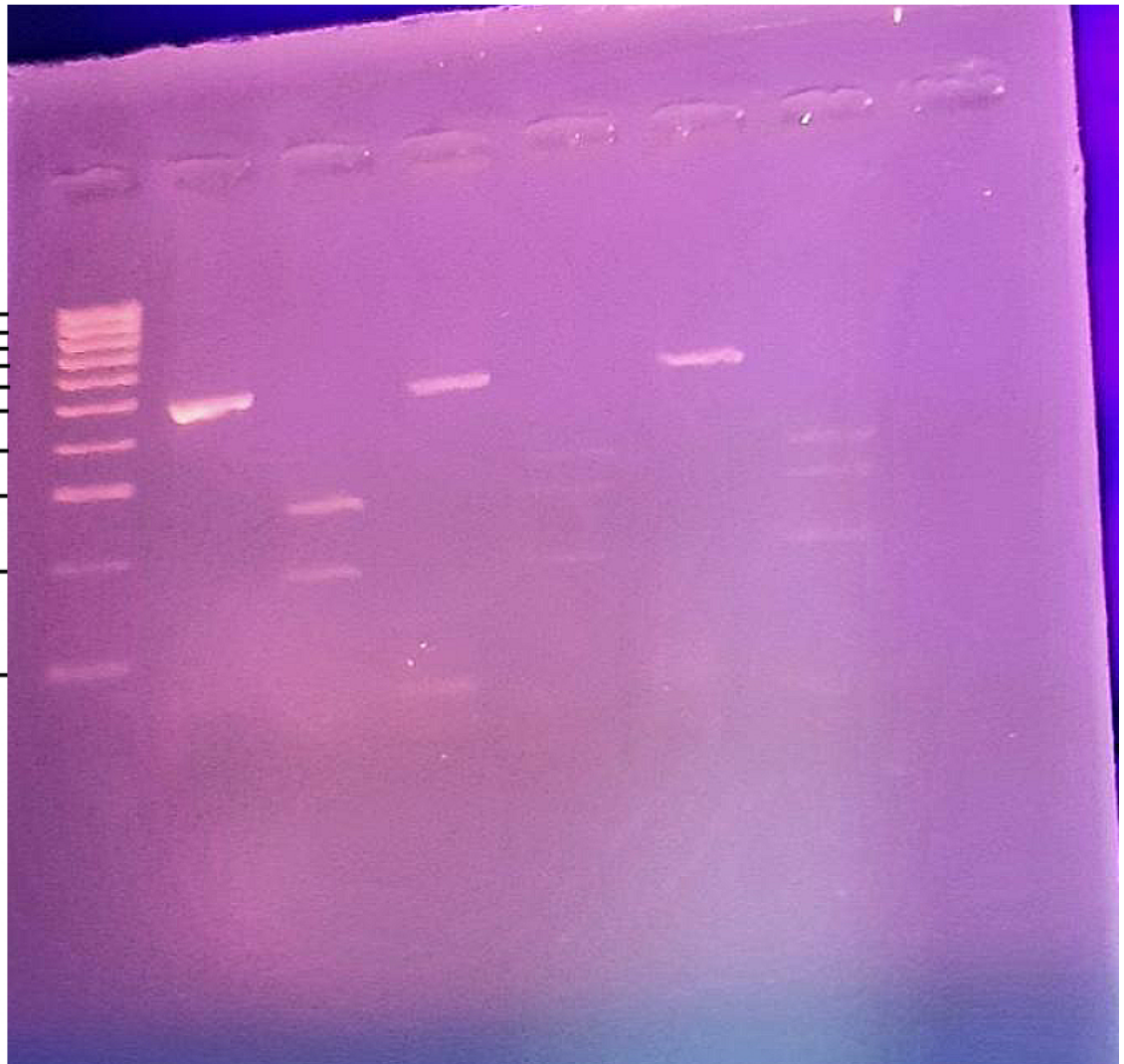

Supplement: S1_raw_images — (PDF) [file pone.0325289.s001.pdf]
